# Assembly of the *β*-Barrel Outer Membrane Proteins in Gram-Negative Bacteria, Mitochondria, and Chloroplasts

**DOI:** 10.5402/2012/708203

**Published:** 2012-11-20

**Authors:** Rajeev Misra

**Affiliations:** School of Life Sciences, Arizona State University, Tempe, AZ 85287, USA

## Abstract

In the last decade, there has been an explosion of publications on the assembly of *β*-barrel outer membrane proteins (OMPs), which carry out diverse cellular functions, including solute transport, protein secretion, and assembly of protein and lipid components of the outer membrane. Of the three outer membrane model systems—Gram-negative bacteria, mitochondria and chloroplasts—research on bacterial and mitochondrial systems has so far led the way in dissecting the *β*-barrel OMP assembly pathways. Many exciting discoveries have been made, including the identification of *β*-barrel OMP assembly machineries in bacteria and mitochondria, and potentially the core assembly component in chloroplasts. The atomic structures of all five components of the bacterial *β*-barrel assembly machinery (BAM) complex, except the *β*-barrel domain of the core BamA protein, have been solved. Structures reveal that these proteins contain domains/motifs known to facilitate protein-protein interactions, which are at the heart of the assembly pathways. While structural information has been valuable, most of our current understanding of the *β*-barrel OMP assembly pathways has come from genetic, molecular biology, and biochemical analyses. This paper provides a comparative account of the *β*-barrel OMP assembly pathways in Gram-negative bacteria, mitochondria, and chloroplasts.

## 1. Introduction

The outer membrane encircles Gram-negative bacteria, mitochondria, and chloroplasts. Embedded within this membrane is a unique class of proteins that fold into a *β*-barrel structure consisting of 8 to 22 antiparallel *β*-strands, interacting through hydrogen bonding to the neighboring strands, with the first strand being frequently hydrogen bonded to the last strand. Atomic structures of an impressive number of the *β*-barrel outer membrane proteins (OMPs) have been solved [1]. In general, the nonpolar side chains of a folded *β*-barrel are oriented outwardly towards the lipid bilayer of the membrane, while the polar side chains are exposed inwardly towards the interior of the barrel that often forms a channel. The two main activities of the *β*-barrel OMPs are to permit transport/insertion of proteins and diffusion of solutes across the outer membrane. The latter class of proteins is called porin [2]. These fundamental activities make the outer membrane an immensely important cellular structure of Gram-negative bacteria and eukaryotes.

In Gram-negative bacteria, the assembly and insertion of porin and non-porin proteins are catalyzed by the *β*-barrel assembly machinery (BAM) [3–6]. The BAM complex also assists in the assembly/insertion of lipopolysaccharide (LPS) transporter [7, 8] as well as the transporter of virulence factors into the outer membrane [9]. Given that the majority of proteins in mitochondria and chloroplasts are nuclear encoded, they must be imported into these organelles from the cytoplasm. The major route of protein import into mitochondria and chloroplasts is through the channel-forming complexes called TOM (translocase of the outer mitochondrial membrane) and TOC (translocon at the outer envelope membrane of chloroplasts), respectively [10, 11]. The core proteins of these complexes are Tom40 and Toc75, which are predicted to fold into *β*-barrels. While TOM and TOC complexes are responsible for protein import, the insertion of *β*-barrel OMPs, including Tom40 and Toc75-III, into the outer membranes of mitochondria and chloroplasts is catalyzed by a separate outer membrane-localized complex, which in mitochondria is called SAM for sorting and assembly machine [12]. SAM is also known as TOB for topogenesis of mitochondrial outer membrane *β*-barrel proteins [13]. The SAM/TOB complex is responsible for the assembly and insertion of all known mitochondrial *β*-barrel OMPs. Although the machinery in chloroplasts responsible for the insertion of *β*-barrel OMPs has not been extensively studied, the available data suggest that a Toc75-III paralog, called Toc75-V or OEP80 [14], catalyzes these events.

Given the bacterial origin of mitochondria [15] and chloroplasts [16], some overlaps between the BAM/SAM/Toc75-V (OEP80)-mediated *β*-barrel assembly pathways are expected. Indeed, this is the case, but to a lesser degree than one would have anticipated [17]. This paper presents a historical account of the studies leading to the discovery of the BAM/SAM/Toc75-V (OEP80) complexes and compares the overlapping and distinctive features of the *β*-barrel OMP assembly pathways in Gram-negative bacteria, mitochondria, and chloroplasts.

## 2. Discovery of the **β**-Barrel OMP Assembly Machinery

Multiple and diverse approaches have led to the discovery of machineries dedicated for the assembly of the *β*-barrel OMPs in bacteria, mitochondria, and chloroplasts. In some instances, serendipity played a role, a not-so-unusual incident in many scientific discoveries, while in other cases, traditional genetic and biochemistry/molecular genetics approaches coupled with genomics/proteomics and bioinformatics were instrumental in discovering the components of the assembly machinery. Although the seeds for the discovery of the *β*-barrel OMP machineries were sown several decades ago, 2002–2005 in particular were bumper years for the *β*-barrel OMP assembly breakthroughs. During this period, Toc75-V/OEP80, Omp85/BamA, and Sam50/Tob55 were identified as the core and essential components of the machineries dedicated to the assembly of the *β*-barrel OMPs in chloroplasts, bacteria, and mitochondria, respectively [18–24].

Two reports in the late nineties provided evidence for evolutionary and functional links between the Toc75 (Toc75-III) protein of the chloroplast and a protein, Slr1227, from the outer membrane of cyanobacteria [25, 26]. The conservation of Slr1227 homologs in other bacterial species, whose genome sequences were known at the time, led to the proposal that they belong to a family of OMPs involved in transport function [26]. These studies provided the framework for subsequent analyses leading to the discovery of the essential machinery dedicated in the assembly of *β*-barrel OMPs. It is important to note that in 2002, Eckart et al. [18] identified a Toc75-III homolog from the outer envelope of pea chloroplasts, Toc75-V, whose sequence resembled more closely with the bacterial homologs than that reported earlier for Toc75-III. Two years after the initial discovery of Toc75-V from pea chloroplasts, through genomics approach, a Toc75-III paralog was identified from the *Arabidopsis thaliana* chloroplast outer envelope and was named AtOEP80, for *A*. *thaliana* Outer Envelope Protein of 80 kDa [23]. Despite these revelations, Toc75-III deserves the credit for realizing the existence of phylogenetically and functionally linked proteins in the outer membrane of Gram-negative bacteria and chloroplasts. Like Toc75-III, Toc75-V/AtOEP80 is essential [27].

The first concrete proof of the function of these conserved transport-related proteins came from the analysis of Omp85 from *Neisseria meningitidis* [21]. The scientists applied the rationale that components of the *β*-barrel OMP insertion machinery are likely to be conserved and essential, and indeed, Omp85, like Slr1227, could not be deleted from the chromosome unless the deletion allele was simultaneously complemented by a plasmid-borne copy of Omp85. Moreover, *N*. *meningitidis* cells depleted for Omp85 displayed anomalous *β*-barrel OMP assembly [21]. The discovery of the Omp85 homolog, BamA, in *Escherichia coli* and accessory proteins of the BAM complex has an interesting beginning involving the use of an in-frame deletion allele of *lptD*. Mutations in *lptD*, originally named *imp* for increased membrane permeability, were first identified among revertants that grew on a minimal medium containing maltodextrin, a polymer of glucose, in the absence of the LamB maltoporin [28]. The ability for *lptD* mutants to grow on maltodextrin as the sole carbon source without LamB was determined to be the result of an increased membrane permeability defect that, besides allowing large sugar molecules to enter the cell, also let large antibiotics, such as bacitracin and rifampin, to infiltrate the outer membrane [28]. Among the *lptD* mutations, the *lptD4213* allele was subsequently used in a genetic selection to better understand the mechanism of resistance to glycolipid derivatives of vancomycin that inhibit transglycosylase activity [29]. Mutations in the *bamB* (*yfgL*) gene were identified among transglycosylase inhibitor resistant colonies in an *lptD4213* background [29]. To obtain further clues as to the cellular role of BamB, pull-down assays were carried out using a His-tagged BamB variant as bait that led to the identification of BamA, BamC, and BamD [24]. BamE was later identified through a similar pull-down/affinity purification analysis [30]. It should be of interest to note that *lptD* turned out to be an essential *E*. *coli* gene [31] that encodes for an outer membrane transporter for LPS [7, 32], an essential outer membrane component in *E*. *coli* [33] but not in *N*. *meningitidis* [34].

Once the BAM complex components were identified, bioinformatics approaches were undertaken to determine their conservation in different bacterial species with known genome sequences. From ClustalW analysis [35] to more comprehensive examination using hidden Markov model [36], it was apparent that BamA and BamD, which are essential in *E*. *coli* [24, 37–39] and *N*. *meningitidis* [21, 40] and perhaps in most, if not all, Gram-negative bacterial species, are the two most conserved proteins of the BAM complex. BamB and BamE are the next two most conserved lipoproteins, followed by BamC [35, 41]. By immuneprecipitating BamA from the detergent-solubilized outer membrane, a new component of the BAM complex, named BamF, was recently identified in *α*-protobacterium *Caulobacter crescentus*, which naturally lacks BamC [36].

The discovery of the SAM complex also has an interesting history. One of the accessory proteins of the SAM complex, Sam37 (also called Mas37 and Tom37), was found serendipitously while screening temperature sensitive yeast mutants to identify genes involved in phospholipid metabolism [42]. As expected, the disruption of the Sam37 (Mas37) gene caused a conditional lethal growth phenotype [42]. Further studies revealed that the Sam37 mutant has reduced phospholipids levels possibly due to an indirect consequence of a defect in the mitochondrial protein import machinery. Based on the genetic and biochemical interaction data, Tom70 (formerly Mas70) and Sam37 were initially thought to function as a heterodimeric receptor complex in the mitochondrial outer membrane [42]. For this reason, Sam37 was also named Tom37 [43, 44]. However, subsequent analyses showed that the two proteins are a part of two separate mitochondrial outer membrane complexes: Tom70 associates with the TOM complex involved in the translocation of mitochondrial proteins, while Sam37 is part of the SAM complex involved in the assembly of the mitochondrial *β*-barrel OMPs [45]. Nevertheless, the initial discovery of Sam37 paved the way for the identification of Sam50 [19] and Sam35 [46], using Sam37 as a bait in affinity purification experiments. Independently, employing the proteomics approach, scientists identified a 55 kDa protein from the mitochondrial outer membrane of *Neurospora crassa* [20]. They named this protein Tob55 in keeping with the TOB nomenclature. Tob38, called Sam35 in the yeast system, was discovered in the same fashion as Sam35, except that the scientists used Tob55 (Sam50) and not Sam37 (Mas37) as a bait in the affinity pulled down assay [47]. Ishikawa et al. [48] also identified Tob38/Sam35, which they called Tom38, by applying the logic that mitochondrial proteins involved in mitochondrial protein assembly/import would be essential. The last known component of the SAM/TOB holocomplex, Mdm10, was discovered via the same affinity purification approach involving the tagged Sam37 [49]. That gene was identified among the temperature sensitive yeast mutants defective in the normal distribution of mitochondria to daughter buds ten years earlier [50].

## 3. A Common Core Component of the **β**-Barrel Assembly Machinery

Omp85/BamA, Sam50/Tob55, and Toc75-V/OEP80 are the core components of the *β*-barrel assembly machineries in Gram-negative bacteria, mitochondria, and chloroplasts, respectively [18–24, 27]. Collectively, they are classified as the Omp85 family of proteins that catalyze insertion and assembly of *β*-barrel OMPs. These essential proteins share a common molecular architecture consisting of an N-terminal soluble domain named POTRA, for polypeptide-transport-associated [51] and a C-terminal transmembrane *β*-barrel domain [21, 52, 53]. Omp85/BamA, Sam50/Tob55, and Toc75-V/OEP80 are also the only components of the *β*-barrel assembly machineries that share a common ancestral origin [52, 53]. Therefore, understanding their structure and the mechanism by which they catalyze the final steps of *β*-barrel OMP assembly has broad significance. The following sections mainly describe Omp85/BamA and Sam50/Tob55, since detailed analysis of Toc75-V/OEP80 is currently lacking. 

## 4. The **β**-Barrel Domain of the Omp85 Family of Proteins

The atomic structure of the C-terminal domain of the Omp85 family of proteins is currently not available. However, it is predicted to fold into a *β*-barrel constructed from 12 [21, 22] or 16 antiparallel *β*-strands [53, 54]. In 2007, the high resolution structure of FhaC, a member of the two-partner secretion system and Omp85/TpsB superfamily, was published [55]. The structure showed that the C-terminus of FhaC folds into a 16-stranded *β*-barrel, the lumen of which is occluded by two substructures, one of which, an N-terminus *α* helix, is absent from the Omp85 family of proteins, while the other, a large loop (loop 6) connecting *β*-strands 11 and 12, contains residues that are highly conserved in the Omp85/TpsB superfamily of proteins [52, 53].

It has been assumed that like FhaC, the lumen of the *β*-barrel of other members of the Omp85/TpsB superfamily will also be occluded by a structure analogous to loop 6. This FhaC loop contains a motif—VRGY/F—conserved in all members of the Omp85/TpsB superfamily [35, 52, 53, 56]. The functional significance of the conserved motif and the loop 6 was first explored in FhaC, where the authors showed that alterations within the conserved motif or deletion of loop 6 impaired or abolished the FhaC-mediated secretion of filamentous haemagglutinin adhesin (FHA) molecules [55, 56]. The first report highlighting the importance of the BamA region corresponding to loop 6 of FhaC came from genetic analysis seeking revertants of an *E. coli* mutant simultaneously lacking BamB and BamE lipoproteins of the BAM complex [35]. Of the six different single amino substitutions identified among revertants that improved growth and OMP phenotypes, four mapped within the hypothetical loop 6 region of BamA and in the vicinity of the conserved VRGF motif. It was hypothesized that BamB and BamE allosterically influence BamA *β*-barrel and its loop 6 region to facilitate OMP assembly. Consequently, without the two Bam lipoproteins, BamA fails to assume a conformationally active state, a defect that is partially reversed by substitutions within BamA's loop 6. Subsequently, site-directed mutagenesis analysis directly tested the hypothesis of the involvement of the conserved “RGF” motif of BamA in *β*-barrel OMP assembly [57]. The data unequivocally showed the importance of the conserved motif not only in the assembly of substrate *β*-barrel OMPs but also of BamA itself [57].

Exactly how the *β*-barrel domain of the Omp85/BamA, Sam50/Tob55, and Toc75-V/OEP80 promotes *β*-barrel assembly remains speculative. As with most *β*-barrel OMPs, BamA/Omp85 and Sam50/Tob55 have been shown to form channels in vitro [20, 58–63]. The channel conductance of BamA/Omp85 has been measured to be between 0.4 and 0.65 nS [58–60, 62], which is similar to the range reported for a Sam50 homolog from *Drosophila melanogaster* [60], but is considerably lower than 3.7 nS reported for in vitro refolded Sam50/Tob55 from *N*. *crassa* [20]. It has been speculated that the large channel diameter of Sam50/Tob55 could accommodate 16–22 *β*-strands [20]. One study put the estimated channel diameter of in vitro refolded BamA/Omp85 to be 25 Å [59], which is almost as large as the protein secreting channel of Tom40 [64]. The in vitro channel activities of BamA/Omp85 could be stimulated in the presence of the denatured *β*-barrel OMPs and a C-terminal peptide derived from an OMP [59]. In contrast, the C-terminal *β*-signal peptide failed to produce any significant effect on Sam50/Tob55 channels but did so on the channel activity of the SAM complex reconstituted in the liposomes [61]. This discrepancy may reflect differences in the ability of BamA/Omp85 and Sam50/Tob55 to recognize/bind to the substrate polypeptides. Apparently, a subunit of the SAM/TOB complex, Sam35/Tob38/Tom38, is required to stimulate the in vitro channel activity of Sam50 [61]. How these in vitro reported channel activities of the Omp85 family of proteins relate to their in vivo function in facilitating insertion and assembly of the *β*-barrel OMPs is not entirely clear.

For protein secretors like FhaC, the challenge is to allow a safe passage for the substrate molecule through their channels across the outer membrane. The FhaC channel diameter has been estimated to be around 3 Å, which is too small to accommodate FHA polypeptides even in their extended conformation. It has been proposed that loop 6, which occludes the barrel lumen, may be dislodged from the channel during the course of FHA secretion [55]. While the expulsion of loop 6, and perhaps the N-terminus *α*-helix 1, is expected to widen the channel diameter from 3 Å to between 8 and 16 Å, these dimensions may still be not large enough to allow the passage of folded FHA molecules. Thus, the final folding of newly secreted FHA molecules is thought to occur after secretion and on the cell surface [55]. In contrast to FhaC, the Omp85 family members mediate insertion and assembly of substrate OMPs in the outer membrane. If the assembling OMPs do indeed enter the channel of a BamA/Omp85 barrel, the channel must eventually open sideways to let the assembling OMPs gain access to the outer membrane. Additional subunits of the assembly machinery, loop 6 of the barrel, and even the substrate molecule might facilitate this event. Alternatively, the assembly and insertion of substrate OMPs occur outside the barrel at the BamA/Omp85-lipid interface or between BamA/Omp85 oligomers, as suggested previously [59]. Regardless of the precise mechanism by which the barrel of the Omp85 family members mediates the final steps of *β*-barrel OMP assembly and insertion, it is apparent from the genetic data that loop 6 plays a critical role in these events. It is also worth noting that in case of FhaC, loop 6 was thought to extend beyond the barrel lumen and into the periplasmic space [55]. If this is also the case with the Omp85 family members, loop 6 would have the opportunity to interact with substrate OMPs and other members of the assembly machinery outside the barrel, besides influencing the channel properties.

## 5. Essentiality and Number of POTRA Domains

Whereas it is generally agreed that the C-terminus *β*-barrel domain of BamA/Omp85/Sam50/Toc75-V/OEP80 likely catalyzes the final step of *β*-barrel OMP assembly and insertion, the role of the N-terminus POTRA domain remains poorly understood and is somewhat controversial.

In bacteria, the POTRA domain of BamA/Omp85 extends into the periplasm, where it is shown to interact with the members of the BAM complex [65] and a major periplasmic chaperone, SurA [8]. Based on the observation that the chromosomal *bamA* gene cannot be deleted from a strain expressing just the C-terminus *β*-barrel domains of BamA, it was concluded that the POTRA domains perform an essential function [58]. Results from another analysis, where the individual POTRA domains of BamA were deleted, one at a time, revealed that POTRA 3, 4, and 5 individually are indispensable; thus they must perform an essential function in *β*-barrel OMP biogenesis. Consistent with this conclusion, our laboratory has recently found that the chromosomal *bamA* gene cannot be deleted from strains expressing plasmid-borne BamA lacking POTRA domains 1 to 4 or 2 to 4 (Leonard-Rivera and Misra, unpublished data). However, a different conclusion was reached for Omp85 of *N*. *meningitidis* where sequential deletion of the POTRA domains revealed that only POTRA 5 is essential in this bacterium [66]. It is likely that differences in the OMP biogenesis pathway in the two bacteria in part may explain the different requirement for the POTRA domains. For example, *N*. *meningitidis* lacks the *E*. *coli* BamB homolog and, unlike in *E*. *coli*, SurA reportedly does not play a major role in OMP biogenesis in *N*. *meningitidis* [67]. Moreover, LPS in *N*. *meningitidis* is dispensable [34], whereas in *E*. *coli,* LPS is not only essential for bacterial viability [33], but it also supports the biogenesis of most *β*-barrel OMPs [68, 69]. These and other genetic and physiological variations between the two bacteria may contribute to differences in the particular POTRA domains required for cell viability.

Unlike in most proteobacteria, the BamA/Omp85 homologs from cyanobacteria and plants, Toc75-V/OEP80, contain three POTRA domains, of which the first and third correspond to the first and fifth POTRA domains of the proteobacterial BamA/Omp85 proteins [63]. No mutational data are currently available for the Toc75-V/OEP80 POTRA domains. Sam50 contains only one POTRA domain, which corresponds to POTRA 5 of BamA/Omp85 [63]. Curiously, the single POTRA domain of Sam50 is found to be dispensable in yeast cells [61, 70]. At present, there are no clearcut answers as to why the number of POTRA domains and their essentiality vary drastically in different organisms/organelles. However, some plausible theories can be proposed. For example, the number of required POTRA domains may correlate roughly with the number of *β*-barrel OMPs the machinery handles in that organism. In other words, the greater the number of substrate *β*-barrel OMPs, the greater the number of POTRA domainsis. In Gram-negative bacteria, it is estimated that between 1.5% and 3% of total open reading frames may encode for *β*-barrel OMPs [71]. Thus, in *E*. *coli* K-12, the number of *β*-barrel OMPs could range from 65 to 130, while in *N*. *meningitidis* their numbers may be between 32 and 64 [70]. A lower number of *β*-barrel OMPs in *N*. *meningitidis* compared to *E*. *coli* could in part explain different POTRA requirements in these two bacteria. The genome size of highly diverse Gram-negative cyanobacteria varies greatly, and it is estimated to encode for a smaller number of *β*-barrel OMPs as a percent of the total number of open reading frames than that of the genomes of *γ* proteobacteria like* E*. *coli* [72]. Consequently, the three POTRA domains found in the *Anabaena sp. *PCC 7120 Omp85 protein [73] may be optimally suited for a relatively smaller number of *β*-barrel OMPs it handles. Similarly, the presence of the solitary POTRA domain in Sam50 is compatible with an estimated 5-6 *β*-barrel OMPs it inserts [72]. 

Bos et al. [66] showed that in an Omp85 POTRA deletion mutant, the assembly of an eight-stranded *β*-barrel OMP was less severely affected than that of a large twenty two-stranded *β*-barrel OMP. Thus, a requirement for certain number of POTRA domains in bacteria at least may also correlate with the number of *β*-strands per barrel and/or the quaternary structure of the *β*-barrel OMPs. Finally, the number and essentiality of POTRA domains may correspond to the complexity of the assembly machinery. For example, machinery that handles a greater number of *β*-barrel OMPs may contain a greater number of accessory proteins. Given that the POTRA domains mediate protein-protein interactions, they are ideally suited not only to interact with substrates OMPs but also with the accessory proteins of the assembly machinery. Indeed, in *E*. *coli*, it has been experimentally demonstrated that the four Bam lipoproteins use POTRA domains 2 to 5 as scaffold to constitute a functional BAM complex [65].

## 6. Role of the POTRA Domain

As the acronym suggests, the POTRA domains were originally predicted to have a chaperone-like function in mediating substrate protein transport across the C-terminus *β*-barrel domain [51]. However, almost a decade after their recognition, the precise roles of the POTRA domains remain somewhat elusive and controversial. The proposed roles include reception/release of substrate OMPs, scaffold for the accessory proteins of the assembly machinery and chaperones, and modulation of the channel activity of the *β*-barrel domain.

Mutagenesis data from BamA/Omp85 and Sam50/Tob55 yielded contrasting data that may reflect evolutionarily distinct roles for the POTRA domains. As mentioned previously, BamA POTRA domains 2 to 5 have been shown to serve as a scaffold for the BamBCDE lipoproteins [65]. Deletion data revealed that BamA POTRA domains 3 to 5 are essential for cell viability in *E*. *coli* [65]. Interestingly, however, only POTRA 5 is required for BamA's interaction with the essential lipoprotein, BamD [65]; hence, the reason for why POTRA 3 and 4 are needed for *E*. *coli* cell viability is not immediately apparent. Deletions of these two domains prevent BamA's interactions with the nonessential lipoprotein, BamB [65], whose absence only modestly affects OMP biogenesis [24, 30, 35, 74, 75]. Thus, essentiality of *E*. *coli* BamA POTRA 3 and 4 domains could be due to their additional activities, such as interactions with the nascent OMPs to help them assemble and/or to coordinate their interactions with BamA *β*-barrel or BamD. Although the POTRA 2 domain of BamA mediates BamA-BamB interactions, its deletion produces little growth and OMP biogenesis defects [65]. In contrast, deletion of POTRA 1, which does not affect BamA's interaction with the Bam lipoproteins, confers a drastic *β*-barrel OMP biogenesis and growth defects [8, 65]. Nevertheless, construction of a strain expressing the BamA POTRA 1 deletion mutant as the sole source of BamA protein is possible by growing cells on a minimal medium where cell doubling time is significantly slower than on a rich medium [8]. Thus, the BamA POTRA 1 domain plays an important role in *β*-barrel OMP biogenesis not involving BamA-BamBCDE interactions.

The POTRA 1 domain has been shown to mediate BamA's interaction with SurA [8, 76], the major periplasmic chaperone involved in the biogenesis of almost all *β*-barrel OMPs in *E*. *coli*, except BamA and TolC [8, 77]. This raises an interesting possibility that the POTRA 1 domain acts as a receptor for the majority of nascent *β*-barrel OMPs being delivered by SurA after their emergence from the Sec translocon. Interestingly, in the POTRA 1 domain deletion background BamA biogenesis is also severely compromised, indicating that POTRA 1 may be involved in receiving nascent BamA molecules [8, 76]. Therefore, a severe OMP biogenesis defect observed in the BamA POTRA 1 deletion mutants is likely the outcome of two independent defects, one resulting from the inefficient reception of nascent *β*-barrel OMPs and BamA molecules and the other, the assembly of a dysfunctional BAM complex, a consequence of the first defect. Consistent with the proposed role of the BamA POTRA domains as receptors for nascent *β*-barrel OMPs, biophysical data exist showing interactions of POTRA domains 1 and 2 with *β*-barrel OMP-derived peptides [78]. Thus, by interacting with both nascent *β*-barrel OMPs and components of the BAM complex, the POTRA domains likely help coordinate the initial steps of the *β*-barrel OMP assembly.

Unlike the presence of multiple POTRA domains in bacterial BamA/Omp85, Sam50/Tob55 of yeast mitochondria contains only one POTRA domain [53]. Although there is some controversy as to the role of this solitary POTRA domain of Sam50/Tob55 in yeast, its absence produces little growth defects at 37°C on media supplemented with a fermentable sugar [61, 70] and a modest defect on media supplemented with a nonfermentable glycerol [70]. Based on the orientation of the Sam50/Tob55 POTRA domain facing the intermembrane space and that *β*-barrel OMP import in mitochondria expressing Sam50/Tob55 POTRA variants partially deleted for the POTRA domain is defective, it was initially concluded that the Sam50/Tob55 POTRA domain recognizes newly imported *β*-barrel OMP precursors [70]. However, subsequent analysis showed that the assembly of the Tom40 precursor, a substrate of the SAM complex, was unaffected in yeast mitochondria expressing a Sam50/Tob55 variant completely deleted for the POTRA domain, thus disputing the proposed “receptor” role for the Sam50/Tob55 POTRA domain [61]. Instead, a more recent report concludes that the Sam50/Tob55 POTRA domain plays a role in the release of *β*-barrel OMP precursors from the SAM complex [79]. Rather, Sam35/Tob38/Tom38, which is exposed to the cytoplasm, has been shown to function as a *β*-barrel OMP receptor in mitochondria [61]. It has been argued that the presence of the residual POTRA sequence in the partial deletion variants of Sam50/Tob55 interferes with the precursor transport function [61], which led to the initial conclusion assigning a receptor role to the POTRA domain [70]. Regardless, it is abundantly clear from studies carried out in bacteria and yeast mitochondria that the POTRA domains interact with substrate *β*-barrel OMPs and/or components of the assembly machinery to facilitate *β*-barrel OMP assembly.

Whereas in bacteria and mitochondria, the BamA/Omp85 and Sam50/Tob55 proteins have their POTRA domains oriented towards the periplasm or intermembrane space, a recent report showed that the POTRA domains of Toc75-III and Toc75-V/OEP80 are exposed to the cytoplasmic side of the chloroplast outer membrane [80]. The flip orientation of the Toc75-V/OEP80 POTRA domains would exclude their involvement in the *β*-barrel OMP assembly from the intermembrane space. It remains to be determined whether, in their cytoplasmic orientation, the POTRA domains assist in the reception of the incoming nascent *β*-barrel OMPs or at a later step of their assembly in the outer membrane or both.

## 7. Accessory Proteins of the **β**-Barrel OMP Assembly Machineries

As mentioned previously, BamA/Omp85, Sam50/Tob55, and Toc75-V/OEP80 are the only components of the *β*-barrel machinery in Gram-negative bacteria, mitochondria, and chloroplasts that share significant homology, indicating a common ancestral origin. In contrast, however, the accessory proteins that interact with these core proteins or substrate OMPs appear to be unique to bacteria or mitochondria. The accessory components that interact with Toc75-V/OEP80 of chloroplasts have not been identified. By accessory proteins, it is not implied that they are dispensable components of the machinery. For example, BamD of the BAM complex of Gram-negative bacteria and Sam35/Tob38/Tom38 of the SAM/TOB complex of mitochondria are essential for cell viability [39, 40, 46, 47].

## 8. Accessory Proteins of the BAM Complex: The Bam Lipoproteins

The BamBCDE lipoproteins, formerly referred to as YfgL, NlpB, YfiO, and SmpA, respectively, together with BamA (YaeT), constitute the BAM holocomplex [24, 30]. Deletion analyses and pull-down assays have shown that BamB interacts with BamA's POTRA domains 2 to 4 independent of BamCDE [65]. BamD forms a subcomplex with BamCE [39], and together their interaction with BamA requires the POTRA 5 domain [65]. Recent evidence suggests the existence of an additional BamCD subcomplex [81]. As mentioned previously, only the accessory protein BamD of *E*. *coli* and its homologs in other bacteria are shown to be essential for cell viability [36, 39, 40, 82, 83], indicating that they carry out an essential role in *β*-barrel OMP assembly. Although BamBCE individually are dispensable for cell viability, their pair wise absence severely compromises cell growth and *β*-barrel OMP biogenesis [30, 35]. This suggests that the nonessential lipoproteins play an overlapping role that is critical for the activity and stability of the BAM complex [35]. Interestingly, compensatory alterations that improve the growth of an *E*. *coli* mutant simultaneously lacking BamB and BamE map within the *β*-barrel domain of BamA [35]. The majority of these alterations reside within the predicted loop 6 region and in the vicinity of the conserved VRGF motif there [35]. An interpretation of this finding is that normally BamB directly and BamE indirectly (via BamD) influence the conformation of the *β*-barrel domain of BamA to stabilize and functionally activate the BamA protein. Consequently, without BamB and BamE, BamA becomes unstable and cannot be fully activated, resulting in synthetic and conditional lethal phenotypes. However, subtle alterations within the *β*-barrel domain of BamA stabilize the protein in the absence of BamB and BamE and allow it to assume a partially active functional conformation [35]. This interpretation is consistent with a recently published paper in which the authors showed that BamE modulates the conformation of BamA [84].

Why is BamD essential? The answer to this question may partly lie in its two-domain structure with two distinct activities [85–87]: the N-terminal domain of BamD is thought to interact with substrate OMPs, while its C-terminal domain has been experimentally shown to be important for interactions with BamA, BamC, and BamE [30, 39]. The structure of BamD shows that it contains all-helical, five tetratricopeptide repeats (TPRs) known for mediating protein-protein interactions [88]. Given that BamD is an outer membrane lipoprotein, its lipid-modified N-terminal end must lie in close proximity to the inner face of the outer membrane, thus closer to the BamA *β*-barrel domain than the soluble POTRA domains that extend into the periplasm. In this orientation, binding of substrate OMPs to BamD's N-terminal domain may either facilitate their delivery to the BamA *β*-barrel domain or reception from it for subsequent assembly/insertion into the outer membrane. A recent crystal structure of BamD complexed with the unstructured N-terminal region of BamC shows that BamC occupies the presumed N-terminal substrate-binding pocket of BamD [89]. Thus, BamC may regulate binding of substrate OMPs to the first three TPR folds of BamD [87, 89]. The essential nature of BamD suggests that it is involved in some critical steps of OMP assembly. Based on the observation that separately isolated BamA and BamD can be reconstituted in vitro into a functional complex, it has been proposed that BamD and BamA function independently but in a coordinated manner [90]. Whether BamD serves as a major conduit of OMP delivery to BamA or acts at a later step of OMP assembly, involving the integration/oligomerization of OMPs in the outer membrane remains to be determined.

In a recent exciting finding, Ricci et al. [91] isolated a novel, gain-of-function BamD mutant in which a substitution, R197L, mapping at the C-terminal domain of BamD, overcomes the functional defect of a BamA POTRA 5 mutant without restoring stable interaction between BamD and the mutant BamA protein. They proposed that the novel BamD mutant has adopted a constitutively activated state that wild type BamD normally assumes upon interaction with the POTRA 5 domain of wild type BamA. Although the precise mechanism by which the mutant BamD protein overcomes the defect of the BamA POTRA 5 mutant or restores OMP assembly remains to be elucidated, it reveals that stable BamA-BamD interactions are not absolutely necessary for normal OMP assembly. Their results further corroborate an idea that BamD and BamA influence OMP assembly by directly interacting with substrate OMPs.

Although BamBCE are not essential lipoproteins in *E*. *coli* or widely conserved like BamA and BamD, their direct or indirect interactions with BamA and BamD likely influence the conformation of these two essential components of the BAM complex and make them more efficient in receiving and assembling substrate OMPs. Mutagenesis data revealed five conserved residues of the mature BamB protein (L173, L175, R176, D227, and D229) to be important for interactions with BamA [92]. In the three-dimensional structure of BamB (see later), these five residues are located in close proximity to each other and form a contiguous patch on the surface of the protein. Further evidence that the two regions of BamB (P171–P181 and E221–D229) interact with or are in close proximity to BamA was provided by site-specific, in vivo cross-linking analysis [92]. Based on the data obtained from POTRA deletion analysis and mutagenesis of a POTRA 3 region that forms a *β*-bulge, BamB likely interacts with POTRA domains 2–4 [65].

The high resolution structures of the three nonessential Bam lipoproteins have also been solved [87, 89, 93–96]. BamB's structure contains an eight-bladed *β*-propeller fold and WD40 motifs known to mediate protein-protein interactions and scaffolding of multiprotein complexes [97]. On the basis of its structure, it has been argued that besides binding to BamA, BamB may also bind to unfolded OMPs and help “channel” substrates to BamA [93]. However, this idea has not been supported by experiments that sought to cross-link amphipathic peptides to BamB in vitro [87]. Alternatively, it is possible that BamB primarily scaffolds BamA and influences the conformation of its POTRA domains to modulate their ability to receive substrate OMPs being delivered by the periplasmic chaperone, SurA [8, 76, 92, 95]. The presence of a hinge point between POTRA 2 and 3 makes these two domains flexible and enables the POTRA domains to adopt two distinct conformations [98]. Thus, binding of BamB to POTRA 2 and 3 is likely to modulate POTRA conformation and activity. The in vitro reconstitution system of the functional BAM complex system further validated the significance of BamB in *β*-barrel OMP assembly [90, 99]. In fact, the absence of BamB from the in vitro system results in a much stronger *β*-barrel OMP assembly defect [90] than that observed in vivo [74, 75, 92], thus further validating a critical role of BamB in *β*-barrel OMP assembly. 

BamCE interact with BamA via BamD and independent of BamB [30, 39]. Consequently, the absence of BamC or BamE reduces BamD's ability to efficiently interact with BamA but results in only a weak OMP assembly defect. However, the simultaneous absence of BamBC produces a strong and synthetic phenotype [30], indicating that they possess some overlapping activities that are critical for BamD's activity and its ability to interact with BamA. As mentioned previously, the simultaneous absence of BamB and BamE, the members of two separate BAM subcomplexes, also produces synthetic and conditional lethal phenotypes, which can be overcome by single amino acid substitutions mapping in the BamA *β*-barrel domain [35]. Interestingly, the same BamA *β*-barrel substitutions also reverse the synthetic phenotypes of cells simultaneously lacking BamCE (Tellez and Misra, unpublished data), indicating a partly overlapping defect resulting from the absence of the accessory components of the two subcomplexes. Based on recently solved three-dimensional structures and mutagenesis data, additional envelope-associated roles have been proposed for BamE, including preferential binding to phosphatidylglycerol [96] and cell wall remodeling [87]. Although further work is needed to better understand these potential activities of BamE, if confirmed, they would shed light on the localization of the BAM complex at preferential sites on the bacterial envelopes and coordinated biogenesis of the outer membrane and cell wall.

BamC is the least conserved lipoprotein of the BAM complex, and a *bamC* null allele produces a weak phenotype [24]. The absence of a BamC homolog in *C*.* crescentus* and other members of the *α*-proteobacteria class has led to a hypothesis that the *β*-barrel OMP assembly machinery in some bacteria may contain novel lipoproteins [36]. Consistent with this hypothesis, a new lipoprotein member of the BAM complex, BamF, was recently identified in *C*.* crescentus* [36]. BamF, which is found exclusively in *α*-proteobacteria, shares a common motif with BamC, and, like BamC, it is not essential for bacterial viability. Additional work will be needed to establish whether BamF is analogous to BamC, and if not, what roles it plays in the BAM complex. A recent paper reported that *E*. *coli* BamC has a unique membrane topology, with its C-terminus being exposed at the cell surface [81]. It is unclear whether a subpopulation of BamC adopts this topology during OMP assembly or that the entire BamC population is permanently present in this state. A relevance of this observation to the functioning of BamC in *β*-barrel OMP assembly remains to be determined.

## 9. Accessory Proteins of the SAM/TOB Complex: Sam35/Tob38/Tom38, Sam37/Mas37, and Mdm10

The mitochondrial SAM/TOB complex has three accessory proteins, Sam35/Tob38/Tom38, Sam37/Mas37, and Mdm10, whose functions are required for proper *β*-barrel OMP assembly. Of the three proteins, only Sam35/Tob38/Tom38 is essential for cell viability in the yeast cell [46–48, 100]. Although Sam37/Mas37 is dispensable for cell viability, mutants lacking this protein display a temperature sensitive growth phenotype [42] and are impaired in *β*-barrel OMP biogenesis [45]. The essential nature of the core Sam50/Tob55 and accessory Sam35/Tob38/Tom38 makes them analogous to the BamA/Omp85 and BamD proteins of the bacterial BAM complex. Both Sam35/Tob38/Tom38 and Sam37/Mas37 are peripheral OMPs with regions exposed to the cytoplasm [46–48], displaying a topology opposite to Sam50/Tob55, with respect to its POTRA domain, and to the BamBDE lipoproteins of the *E*. *coli* BAM complex, but similar to that recently reported for BamC [81]. 

Three reports in 2004 showed that Sam35/Tob38/Tom38 aids in Tom40 biogenesis by influencing the formation of the assembly intermediate I, where it, together with Sam50/Tob55 and Sam37/Mas37, interacts with Tom40 [46–48]. In addition to Tom40, biogenesis of other *β*-barrel OMPs, such as Sam50/Tob55 and Mdm10, but not that of non-*β*-barrel proteins present in the mitochondrial outer membrane or other subcompartments of mitochondria, was also affected in yeast cells either depleted for Sam35/Tob38/Tom38 [47, 48] or expressing a mutant form of Sam35/Tob38/Tom38 from a temperature sensitive allele [46]. Moreover, it was revealed that Sam35/Tob38/Tom38 interacted directly with the assembly intermediates of Tom40 and Mdm10 independent of Sam37/Mas37 [47]. Thus, Sam50/Tob55 and Sam35/Tob38/Tom38 form the core unit of the SAM/TOB complex, with which Sam37/Mas37 and Mdm10 interact dynamically to facilitate the assembly of *β*-barrel OMPs in mitochondria.

A genetic analysis led to the isolation of several temperature sensitive alleles of *sam35* that were useful in dissecting the roles of Sam35/Tob38/Tom38 and Sam37/Mas37 in the *β*-barrel OMP assembly process [101]. Detailed examination of two such *sam35* alleles, whose temperature sensitivity phenotypes were reversible by Sam37/Mas37 overexpression, revealed that one of them affected the activity of Sam35/Tob38/Tom38 and the SAM/TOB complex in substrate binding, while the other affected steps subsequent to substrate binding. This led to the proposal that Sam35/Tob38/Tom38 likely functions as the receptor of the substrate OMPs, while Sam37/Mas37 acts to stabilize Sam35/Tob38/Tom38 and helps release the substrate molecules bound to the SAM/TOB complex [101]. The proposed role of Sam37/Mas37 is consistent with Wiedemann et al.'s observation that the initial import of a SAM/TOB substrate OMP, Tom40, was not significantly affected in the absence of Sam37/Mas37, but subsequent steps leading to the formation of the TOM complex were severely impaired [102].

Bioinformatics and experimental evidence indicated that Sam35/Tob38/Tom38 recognizes a C-terminus proximal “*β*-signal” found in all four families of the mitochondrial *β*-barrel OMPs [61]. This finding was confirmed by the observation that alterations affecting the conserved residues within the *β*-signal inhibited interaction between the *β*-barrel OMP assembly intermediates and the SAM/TOB complex and/or their subsequent insertion into the outer membrane and maturation [61]. The assembly and insertion of *β*-barrel OMPs have been shown to occur from the *trans* (intermembrane space)-side of the mitochondrial outer membrane [103]. As mentioned earlier in this paper, the single POTRA domain of Sam50/Tob55 is exposed in the intermembrane space and thus would be a logical candidate site for initial substrate recognition/binding. Remarkably, however, deletion of the entire POTRA domain of Sam50/Tob55 failed to prevent the formation of the well-documented assembly intermediates of Tom40 [103], a substrate of the SAM/TOB complex, indicating that the POTRA domain of Sam50/Tob55 is not involved in the initial substrate recognition/binding [61]. Instead, it has been recently shown that the POTRA domain plays a role in the release of substrate OMPs from the SAM/TOB complex [79]. Consequently, Sam35/Tob38/Tom38 is now thought to be the primary receptor of *β*-barrel OMP assembly intermediates. If, however, the majority of Sam35/Tob38/Tom38 is exposed to the cytosolic side of the outer membrane, how then does it recognize the assembly intermediates being presented to it from the *trans*-side of the mitochondrial outer membrane? This has led to the re-examination of the Sam35/Tob38/Tom38 topology that was originally determined using N- or C-terminally-tagged variants of the protein [46–48]. The new topology data from the untagged Sam35/Tob38/Tom38 protein suggests that it is embedded within the mitochondrial outer membrane in a proteinaceous environment and in close proximity to Sam50/Tob55 [61]. Based on this, Kutik et al. [61] hypothesized that Sam35/Tob38/Tom38 may be present within the oligomeric ring of Sam50/Tob55. It is unclear whether a domain of Sam35/Tob38/Tom38 extends into the Sam50/Tob55 channel to receive the *β*-barrel OMP intermediates for subsequent assembly or that OMP intermediates, via their last *β*-strands, reach out to Sam35/Tob38/Tom38 through the Sam50/Tob55 channel.

Further work is needed to clarify Sam35/Tob38/Tom38's topology relative to Sam50/Tob55 and establish exactly how it makes initial contacts with the newly translocated nascent *β*-barrel OMPs. Moreover, the underlying mechanism of substrate release from the SAM/TOB complex by Sam50's POTRA domain and Sam37/Mas37 remains ill defined. The fact that overexpression of Sam37/Mas37 cannot reverse the growth phenotype of a yeast strain expression Sam50/Tob55 lacking its POTRA domain indicates that Sam37/Mas37 and the POTRA domain of Sam50/Tob55 play a distinct role in the assembly of mitochondrial *β*-barrel OMPs [79].

As stated earlier, *mdm10* was discovered in a screen for temperature sensitive yeast mutants defective in normal mitochondrial distribution and morphology [45]. In 2004, an unexpected discovery of Mdm10 as a part of the SAM complex led to the idea that it is also involved in some aspect of *β*-barrel OMP assembly [49]. Mdm10, like Tom40, Sam50/Tob55, and the porin protein, has been assumed to fold into a *β*-barrel structure. Consistent with this notion, its assembly is dependent on the intact SAM/TOB complex [45]. The initial report showed that Mdm10 plays a specific role in the late steps of the assembly of the TOM complex involving association of Tom40 with Tim22 and small Tom proteins [45]. A subsequent analysis, however, questioned the use of null alleles to dissect the role of Mdm10 because of the reported involvement of Mdm10 in lipid biosynthesis, via its association with the endoplasmic reticulum-mitochondria tethering complex composed of Mmm1, Mdm10, Mdm12, and Mdm34 proteins [104]. Consequently, in an *mdm10* null background or a background severely depleted of Mdm10, a defect in lipid synthesis is likely to result in a pleiotropic phenotype, thus complicating the ability to tease out a specific role of Mdm10 in the SAM/TOB complex. To avoid this pleiotropic effect, Yamano et al. [105] constructed an ingenious Mdm10 mutant with a defective *β*-signal, which was impaired in its ability to interact with the SAM complex but synthesized normal amounts of lipids presumably due to normal interactions with the Mmm1/Mdm12/Mdm34 complex. An examination of the TOM complex assembly in this Mdm10 mutant background revealed that an early step of assembly involving the dissociation of Tom40 from the SAM/TOB complex, that is, the conversion of the assembly intermediate I to II, was defective [105]. Although both Mdm10 and Sam37/Mas37 appear to catalyze the same assembly step of releasing the substrate OMPs from the SAM/TOB complex, Mdm10 displays specificity towards Tom40 [49, 105], while Sam37/Mas37 plays a broader role in the biogenesis of Tom40, porin, and Mdm10 [45, 101]. In spite of this difference, both Mdm10 and porin appear to compete for the same binding site in the SAM/TOB complex as the absence [49] or overexpression [105] of Mdm10 leads to increase or decrease in porin assembly.

## 10. The **β**-Barrel OMP Assembly Pathways

**Figure 1:**
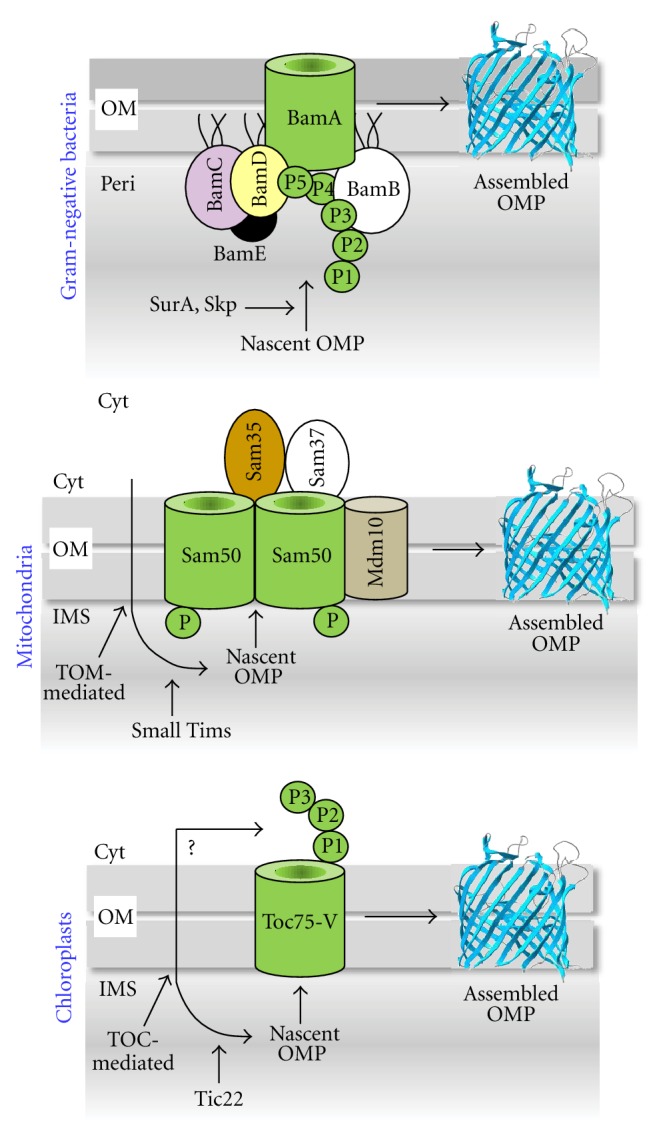
Models of *β*-barrel OMP assembly in Gram-negative bacteria, mitochondria, and chloroplasts. In Gram-negative bacteria, such as *Escherichia coli*, the outer membrane proteins (OMPs) are synthesized in the cytoplasm (Cyt) as precursors with an N-terminal signal sequence. Precursor OMPs exit the cytoplasm via the inner membrane-localized Sec translocon (not shown). Cleavage of the signal sequence during the translocation process leads to the transient appearance of the mature nascent OMPs in the periplasm (Peri) where they interact with chaperones, such as SurA and Skp. The chaperones-bound OMPs are then offloaded to the BAM complex, which assembles and inserts them into the outer membrane (OM) as *β*-barrel proteins. Like in bacteria, OMPs are synthesized in the cytoplasm of eukaryotic cells. From there, OMPs are imported into the intermembrane space (IMS) via the TOM (mitochondria) and TOC (chloroplasts) complexes. In the IMS-side of the mitochondrial outer membrane, small Tim chaperones interact with the nascent OMPs and help their transfer to the SAM complex for assembly and insertion into the outer membrane as *β*-barrel proteins. The core component of the mitochondrial TOM complex, Tom40, is also a *β*-barrel OMP, whose biogenesis is dependent on additional assembly factors, including Mdm10, another *β*-barrel OMP. In case of chloroplasts, it is unclear whether after synthesis in the cytoplasm the nascent *β*-barrel OMPs are first imported into the IMS via the TOC complex for subsequent assembly or from the cytoplasm they interact directly with the Toc75-V, whose three POTRA domains are recently shown to be oriented towards the cytoplasm. Like small Tims of the mitochondrial IMS, Tic22 of chloroplasts may chaperone nascent *β*-barrel OMPs in the IMS if indeed they follow the IMS pathway. The oligomeric states of BamA, Sam50, and Toc75-V are arbitrarily drawn. See text for alternative protein/complex names. P and P1 to P5 denote the POTRA domains.

The journey of OMPs towards their final destination begins in the cytoplasm where they are synthesized (Figure 1). The precursor OMPs are recognized by cytoplasmic chaperones and targeting factors that guide them to the channel-forming translocons—Sec, TOM, and TOC—of bacteria, mitochondria and chloroplasts, respectively. These translocons are localized in the inner membrane in bacteria and the outer membrane in mitochondria and chloroplasts. Consequently, nascent OMPs in bacteria approach the *β*-barrel OMP assembly machineries in one direction, whereas those in mitochondria and chloroplasts would be expected to approach the assembly sites in the opposite direction. However, the discovery of a mitochondrial *β*-barrel OMP assembly intermediate exposed to the intermembrane space [103] has led to the proposal that nascent OMPs in mitochondria also approach the assembly machinery (SAM/TOB) from the same direction as in bacteria (Figure 1). Although in chloroplasts the *β*-barrel OMP assembly pathways are not yet well defined, a recent publication provided evidence that the POTRA domains of Toc75-V/OEP80 are exposed to the cytoplasmic side of the outer membrane [80], thus in reverse orientation to that of BamA/Omp85 and Sam50/Tob55. It is not known whether this altered topology of Toc75-V/OEP80 would mean that in chloroplasts precursor/nascent *β*-barrel OMPs approach the assembly machinery directly from the cytoplasm side, or like in mitochondria, they approach from the intermembrane space side (Figure 1). The latter scenario is supported by a recent publication that suggests the involvement of Tic22, a chaperone-like protein that resides in the intermembrane space of chloroplasts [106] in the assembly of *β*-barrel OMPs in cyanobacteria and plants [107].

Chaperones present in the bacterial periplasmic or intermembrane space of mitochondria and chloroplasts would interact with the nascent *β*-barrel OMPs to keep them in a folding/assembly competent state and assist their handoff to the BAM/SAM/Toc75-V (OEP80) machineries (Figure 1). Of the two major periplasmic chaperones, SurA and Skp, SurA in *E*. *coli* plays the primary role in *β*-barrel OMP assembly [108–111], while Skp is reported to be the major player in *N*. *meningitidis* [67]. It is worth mentioning that not all *E. coli* *β*-barrel OMPs, including BamA and TolC, are dependent on SurA or Skp [8, 112]. It is thought that the intrinsic folding properties of these two proteins, which contain a large soluble domain composed of *α*-helices (TolC) or mixed *α*-helices and *β*-strands (BamA), render their folding independent of any known periplasmic chaperone [8, 113]. Four small Tim chaperones in the mitochondrial intermembrane space form two independent heterooligomeric complexes, Tim8-Tim13 and Tim9-Tim10, and are thought to be functionally analogous to SurA/Skp in the bacterial periplasm [114]. Similarly, Tic22, present in the intermembrane space of chloroplasts [106], is thought to be functionally analogous to SurA and small Tims [107].

The available data suggest that different components of the outer membrane-bound assembly machineries in bacteria and eukaryotes receive chaperone-bound nascent OMPs. SurA has been shown to interact with BamA [92, 110] and, more specifically, with the POTRA 1 domain of BamA [8, 76]. This suggests that POTRA 1 of BamA serves as one of the major docking sites for SurA-bound OMPs. Consistent with this notion, deletion of the POTRA 1 domain, which does not disrupt BamA's interaction with the Bam lipoproteins [65], severely impairs *β*-barrel OMP assembly [8, 65]. The offloading of SurA-bound OMPs to the POTRA domain is thought to occur via *β*-augmentation [8, 78, 98], where the *β*-strands of substrate OMPs align with the *β*-strands of the POTRA domain in a sequence and orientation independent manner [115]. BamB, through its interactions with the BamA POTRA domains, may facilitate transfer of SurA-bound OMPs to the BamA POTRA domains [90, 92, 116]. The fact that the BamA POTRA 1 domain is dispensable for cell viability suggests that secondary docking sites for SurA-OMP may also exist. The POTRA domains 2 and 3, which serve to scaffold BamB, may fulfill this role [78, 98].

Like BamA, the POTRA domain of Sam50/Tob55 was initially thought to serve as the receptor of nascent OMPs being delivered by small Tim chaperones [70]. However, subsequent analysis indicated that Sam35/Tob38/Tom37 may be the primary receptor of nascent OMPs instead [61]. If the nascent OMPs in the intermembrane space are indeed received by Sam35/Tob38/Tom37, then a domain of Sam35/Tob38/Tom37 might be expected to transiently or permanently extend into the intermembrane space side of the outer membrane. However, evidence unambiguously supporting this scenario is lacking. Other than Toc75-V/OEP80 and possibly Tic22, no other components of the machinery dedicated for the *β*-barrel OMP assembly in chloroplasts have been reported so far. A recent in vitro demonstration that *β*-barrel OMPs from chloroplasts can be correctly incorporated into the mitochondrial outer membrane via the TOM-SAM/TOB pathways supports the idea that *β*-barrel OMPs in mitochondria and chloroplasts follow a conserved assembly pathway [117].

After the chaperone-mediated delivery of nascent OMPs to the BamA POTRA domains or Sam35/Tob38/Tom37, it is unclear how and where they assemble into a *β*-barrel structure. As previously discussed, the in vitro channel properties of BamA/Omp85 have been shown to increase in the presence of unfolded *β*-barrel OMPs or peptides corresponding to the last *β*-strands of bacterial OMPs [59]. It is not known whether this increase indicates entry of OMP *β*-strands into the channel formed by a single or multiple BamA/Omp85 *β*-barrels, or destabilization of the BamA/Omp85 *β*-barrel/channel structure. In contrast to BamA/Omp85, fluctuation in Sam50/Tob55 channel properties by the *β*-signal peptides is observed only in the presence of Sam35/Tob38/Tom37, which is consistent with the notion that Sam35/Tob38/Tom37 serves as the primary receptor for the nascent OMPs [61]. It has been hypothesized that a domain of Sam35/Tob38/Tom37 is present inside the central cavity formed by the oligomeric ring of Sam50/Tob55 where it interacts with nascent OMPs via their *β*-signals [61]. Notably missing from these analyses is the role of a conserved loop 6, which, like in FhaC, may also occlude the channel of BamA/Omp85, Sam50/Tob55, and Toc75-V/OEP80. Importantly, recent genetic data have highlighted the significance of loop 6 in the *β*-barrel OMP assembly, including that of BamA itself [35, 57]. The loop 6 may play a direct role if nascent OMPs access channels formed by the individual BamA/Omp85, Sam50/Tob55, and Toc75-V/OEP80 *β*-barrels, but would be involved indirectly if the central cavity formed by their oligomers is the site of assembly.

Several models have been proposed that attempt to capture the steps of *β*-barrel OMP assembly and integration into the outer membrane. It is important to note that only BamA/Omp85, Sam50/Tob55, and Toc75-V/OEP80 are the conserved components of the *β*-barrel OMP assembly machineries in Gram-negative bacteria, mitochondria, and chloroplasts. Moreover, even these conserved components have variable features, for example, they carry different numbers of POTRA domains with reportedly distinct functions, topology, and importance. Therefore, while the general principles of assembly and insertion of *β*-barrel OMPs are expected to be very similar in bacteria and eukaryotes, mechanistically these steps are likely to be achieved quite differently in different assembly systems.

As stated earlier, one model envisions assembly of an OMP *β*-barrel within the proteinaceous environment created by the BAM/SAM/Toc75-V/OEP80 complex. The support for this model comes from electron microscopy studies where scientists observed ring-shaped assemblies of native and recombinant Sam50/Tob55 [20]. These assemblies contained a central cavity with a diameter of 4-5 nm, which is apparently large enough to accommodate 16–22 *β*-strands [20]. Growth of the *β*-barrel by the sequential addition of *β*-strands would induce dissociation of the oligomeric assemblies, followed by the lateral release of fully or partially assembled *β*-barrel into the outer membrane. Given that release of the assembling *β*-barrel into the outer membrane is expected to be an essential step, since without it the central cavity would be jammed, factors that facilitate this release step are also expected to be essential. BamD of the BAM complex fits this criterion. Although Sam37/Mas37 and Mdm10 are implicated in the release step and they are not essential individually, their simultaneous absence leads to synthetic and conditional lethal phenotypes [118], indicating partly overlapping activities. Moreover, the POTRA domain of Sam50/Tob55 has also been reported to influence the release step [79]. Therefore, it appears that in mitochondria multiple factors catalyze the late steps of *β*-barrel OMP assembly. It is worth noting that factors like Mdm10 and Mim1/Tom13 are reported to have more specialized role in the biogenesis of Tom40 [47–49, 105].

In the second model, each individual heterooligomeric complex would play a central role by serving as scaffold for *β*-barrel OMP assembly. The POTRA domains of Gram-negative bacteria and perhaps also in chloroplasts may play a more dominant role in this model than that envisioned in the “central cavity” model earlier. Accordingly, the POTRA domains with the assistance of chaperones (SurA/Skp) and accessory proteins (e.g. BamB) would serve as template for *β*-barrel assembly which would occur close to the membrane interface. The subsequent insertion of the newly assembled barrel would be catalyzed by the BamDCE subcomplex, which coordinates its activity with BamA through interaction with the essential POTRA 5 domain of BamA. This model is consistent with the data collected in numerous in vitro *β*-OMP folding and insertion studies which suggest that chaperones and other proteins of the BAM complex would act as folding and membrane insertion catalysts [119]. The experimental evidence indicates that BamA *β*-barrel does not merely serve to anchor the POTRA domains in close proximity to the outer membrane [66]. As stated earlier, BamA's conserved loop 6, which is predicted to fold inside the barrel, has been experimentally shown to be important for OMP biogenesis in general [35, 57]. The BamA *β*-barrel domain and loop 6 may influence insertion of *β*-barrel into the outer membrane. It is not known what roles BamA/Omp85 channels play in the assembly process. The estimated diameter of BamA/Omp85 channels is thought to be too small to accommodate multiple *β*-strands. A single *β*-strand can enter the channel, but its releases into the outer membrane would require opening of the BamA/Omp85 *β*-barrel, thus temporarily exposing unsatisfied hydrogen bonds in the lipid phase, an energetically unfavorable scenario. An interesting variation outlined by Kim et al. [6] envisions substrate *β*-strands use BamA/Omp85 *β*-strands as folding templates. In other words, substrate OMPs temporarily open and fuse with the BamA/Omp85 *β*-barrel before being released into the lipid bilayer.

## 11. Concluding Remarks

Although a lot of progress has been made in the last decade leading to the identification of *β*-barrel OMP machineries and resolution of atomic structures of many machinery components, much remains to be learned as to how these components/machineries facilitate folding and insertion of *β*-barrel OMPs. The next decade will be an exciting period as the field is poised to gain a deeper understanding of the assembly and discovering additional components of the plant *β*-barrel OMP assembly machinery. Two questions in particular will receive a great deal of attention: where and how does a nascent OMP molecule assemble into a complete *β*-barrel, and how does the newly formed barrel dissociate from the assembly machinery and integrate into the lipid bilayer? Answering these and other relevant questions will require multidisciplinary approaches. One of the important achievements in recent years has been the successful in vitro reconstitution of the functional BAM complex [90, 99]. This opens the door for biochemical testing of various mutants isolated through classical genetics or directed methods and thus allowing data corroboration.

While the core component of the *β*-barrel OMP assembly machineries is preserved in bacteria and eukaryotes, there are significant diversities in the makeup of the accessory components [41], which would indicate species and organelle-specific evolution of the assembly pathways. These variations are likely evolved and optimized for physiology and ecology of the organism. Strikingly missing from the studies involving *β*-barrel assembly pathways is the use of inhibitors, which in other instances have proven to be a powerful tool for dissecting cellular pathways. These inhibitors, when combined with the knowledge of pathway diversities, can be developed into effective and novel antibiotics.
